# Striatal Dopaminergic Innervation Regulates Subthalamic Beta-Oscillations and Cortical-Subcortical Coupling during Movements: Preliminary Evidence in Subjects with Parkinson’s Disease

**DOI:** 10.3389/fnhum.2016.00611

**Published:** 2016-12-06

**Authors:** Andrea Canessa, Nicolò G. Pozzi, Gabriele Arnulfo, Joachim Brumberg, Martin M. Reich, Gianni Pezzoli, Maria F. Ghilardi, Cordula Matthies, Frank Steigerwald, Jens Volkmann, Ioannis U. Isaias

**Affiliations:** ^1^Department of Neurology, University Hospital and Julius-Maximilian-UniversityWuerzburg, Germany; ^2^Department of Nuclear Medicine, University Hospital and Julius-Maximilian-UniversityWuerzburg, Germany; ^3^Centro Parkinson, ASST G. Pini-CTOMilan, Italy; ^4^Department of Physiology, Pharmacology and Neuroscience, CUNY Medical SchoolNew York, NY, USA; ^5^Department of Neurosurgery, University Hospital and Julius-Maximilian-UniversityWuerzburg, Germany

**Keywords:** beta oscillations, motor control, movement disorders, imaging, Parkinson’s disease, subthalamic nucleus, coherence analysis

## Abstract

Activation of the basal ganglia has been shown during the preparation and execution of movement. However, the functional interaction of cortical and subcortical brain areas during movement and the relative contribution of dopaminergic striatal innervation remains unclear. We recorded local field potential (LFP) activity from the subthalamic nucleus (STN) and high-density electroencephalography (EEG) signals in four patients with Parkinson’s disease (PD) off dopaminergic medication during a multi-joint motor task performed with their dominant and non-dominant hand. Recordings were performed by means of a fully-implantable deep brain stimulation (DBS) device at 4 months after surgery. Three patients also performed a single-photon computed tomography (SPECT) with [^123^I]N-ω-fluoropropyl-2β-carbomethoxy-3β-(4-iodophenyl)nortropane (FP-CIT) to assess striatal dopaminergic innervation. Unilateral movement execution led to event-related desynchronization (ERD) followed by a rebound after movement termination event-related synchronization (ERS) of oscillatory beta activity in the STN and primary sensorimotor cortex of both hemispheres. Dopamine deficiency directly influenced movement-related beta-modulation, with greater beta-suppression in the most dopamine-depleted hemisphere for both ipsi- and contralateral hand movements. Cortical-subcortical, but not interhemispheric subcortical coherencies were modulated by movement and influenced by striatal dopaminergic innervation, being stronger in the most dopamine-depleted hemisphere. The data are consistent with a role of dopamine in shielding subcortical structures from an excessive cortical entrapment and cross-hemispheric coupling, thus allowing fine-tuning of movement.

## Introduction

The functional interaction of cortical and subcortical brain areas during movement planning and execution, and in particular the role of striatal dopaminergic innervation, remains unclear. Subjects with Parkinson’s disease (PD) may represent a putative *in vivo* model of dopaminergic denervation (Simuni and Pahwa, 2009) and, when implanted with subthalamic nucleus deep brain stimulation (STN-DBS), can provide the remarkable opportunity to investigate cortical-subcortical interactions by simultaneous recording of local field potential (LFP) and high density electroencephalography (EEG).

STN recordings reveal that when at rest, unmedicated PD patients show an excessively synchronized neuronal activity in the STN and an exaggerated coupling between the STN and the motor cortices (MC). This abnormal activity and coupling is particularly strong in the beta frequency range (≈13–35 Hz) and is reduced by dopaminergic drugs or STN-DBS (Williams et al., 2002; Fogelson et al., 2006; Doyle Gaynor et al., 2008; Kühn et al., 2008; de Solages et al., 2010; Giannicola et al., 2010; Litvak et al., 2011a, 2012; Hirschmann et al., 2013; Kato et al., 2015; Quinn et al., 2015; Weiss et al., 2015; Oswal et al., 2016) and modulated by voluntary movements (Marsden et al., 2001; Cassidy et al., 2002; Lalo et al., 2008; Hirschmann et al., 2013). In particular, movement execution and imagination are associated with beta power changes (Cassidy et al., 2002; Kühn et al., 2004), starting with a decrease or desynchronization in the pre-movement period (event-related desynchronization, ERD) followed by a rebound after movement termination (event-related synchronization, ERS; Cassidy et al., 2002). A similar dynamic pattern of movement-related beta modulation is also present at the cortical level with a morphology that does not substantially differ from that recorded from control subjects (Soikkeli et al., 1991; Alegre et al., 2005; Devos et al., 2006; Meziane et al., 2015; Moisello et al., 2015). Moreover, excessive cortical beta power at rest has been recently correlated with greater movement-related beta-modulation and motor performances (Heinrichs-Graham and Wilson, 2016).

It is likely that striatal dopamine loss is the main cause of abnormal STN activity and cortical-subcortical dynamics in PD (for review, Jenkinson and Brown, 2011; Brittain and Brown, 2014), but direct evidence for this hypothesis is still lacking. We envision a role for dopamine in shielding subcortical structures from excessive cortical drive (Jenkinson and Brown, 2011; Oswal et al., 2013), thus allowing the correct set up of motor programs required for subsequent motor action (Wilson, 2014). A lack of striatal dopaminergic tone, as in patients with PD, would therefore facilitate the basal ganglia entrainment in excessively synchronized oscillatory activity, thus impairing the processing of motor commands (Jenkinson and Brown, 2011; Wilson, 2014). To further elucidate the role of dopamine in cortical-basal ganglia motor processing, we measured beta ERD and ERS, subcortical and cortical-subcortical coherency in patients with PD during a multi-joint, externally-triggered motor task performed with the dominant and non-dominant hand.

Importantly, we determined the level of dopaminergic striatal innervation with a [^123^I]N-ω-fluoropropyl-2β-carbomethoxy-3β-(4-iodophenyl)nortropane (FP-CIT) and single-photon computed tomography (SPECT; Isaias et al., 2010, 2011). Also of relevance, in this study we used an investigational DBS device (Activa PC+S^®^, Medtronic, PLC) that offers the possibility of recording LFPs in the STN in chronically-implanted patients months after surgery.

## Materials and Methods

### Subjects

We tested seven right-handed patients with PD (6 males, 1 female; median age 61 years [range: 67–53 years]; median disease duration 11 years [range: 10–19 years]). All patients were diagnosed according to the UK Parkinson Disease Brain Bank criteria (Hughes et al., 2002) and evaluated with the Unified Parkinson Disease Rating Scale motor part (UPDRS-III). All subjects were right-handed as assessed by a modified Edinburgh handedness inventory. Patients were implanted at the University Hospital of Würzburg between December 2013 and May 2014 with the Activa PC+S^®^ neurostimulation system (Medtronic, PLC). This system allows therapeutic DBS as well as on-demand LFP recordings from the implanted STN electrodes (Rouse et al., 2011; Stanslaski et al., 2012). The Activa PC+S^®^ system and the related hardware and software for programming and readout were provided under a request for application agreement by Medtronic, PLC. The company had no impact on study design, patient selection, data analysis, or reporting of the results. All patients had been selected based on established criteria for DBS surgery (Pollak, 2013). Of relevance, none of the subject had cognitive decline or mood disturbances as evaluated using the Parkinson neuropsychometric dementia assessment (PANDA), Mattis Dementia Rating Scale (MDRS), Hamilton Depression Rating Scale (HDRS) and the Non-Motor Symptoms Scale (NMSS).

The surgical procedure has been described elsewhere (Steigerwald et al., 2008). In brief, implantation was performed under local anesthesia using Leksell’s Frame (Elekta, Leksell Stereotaxy System, Stockholm, Sweden). The DBS electrode used was model 3389 (Medtronic, PLC) with four platinum–iridium cylindrical contacts of 1.5 mm each and a contact-to-contact separation of 0.5 mm. Contact 0/8 was the lowermost and contact 3/11 the uppermost (E0–3 refers to right- and E8–11 to the left-hemisphere). The intended coordinates for STN were 12 mm lateral, 2 mm posterior, 4 mm ventral to the mid-commissural point and were adjusted according to individual STN delineation on T2-weighted and SWI images (Magnetom Trio, Siemens Healthcare, Erlangen, Germany) and with intraoperative microelectrode recordings. Micro- and macro-electrode stimulation and intraoperative CT scan also served to confirm targeting. Postoperative scanning (1 mm slice-thickness, CT scan fusion with the pre-operative MRI) confirmed electrode location. Of note, the presence or absence of LFP power in the beta band was not used to determine the placement of the DBS lead (Quinn et al., 2015).

The precise localization within the STN of the active contacts used for chronic stimulation was further confirmed by image fusion of a non-stereotactic postoperative CT with the preoperative planning MRI by means of Optivise^®^ software under a research agreement with Medtronic, PLC. Correct placement of the DBS electrode was also verified by the clinical response to DBS (meds-off/stim-on) compared to the preoperative improvement of the UPDRS motor score (UPDRS-III) during levodopa challenge (meds-off vs. meds-on; Table 1). Therapeutic response to DBS or levodopa was expressed as percentage of improvement, according to the formula: ((a—b)/a) × 100 (adapted from Isaias et al., 2008) where a = meds-off UPDRS-III score and b = meds-on UPDRS-III at pre-DBS or b = meds-off/stim-on UPDRS-III at the time of the test, 4 months after surgery (post-DBS). The mean percentage of improvement was 65.77% (range: 42.5%–92.72%) due to dopaminergic medication and 68.82% (range: 52.5%–83.63%) due to STN stimulation, thus further supporting correct placement of the DBS electrodes.

**Table 1A:** **Sample characteristics**.

Subject	Gender	Age at surgery (year)	Disease duration at surgery (year)	LEDD pre-DBS (mg)	UPDRS pre-DBS meds-off (score)	UPDRS pre-DBS meds-on (score)	LEDD post-DBS (mg)	UPDRS post-DBS meds-off, stim-on (score)
wue2*	male	65	10	1100	40	23	800	19
wue3	male	61	18	2725	40	9	600	13
wue5*	male	67	17	1050	49	24	500	13
wue6	male	51	11	1133	47	12	180	9
wue7	male	61	10	650	43	24	220	19
wue9*	male	55	19	1200	50	11	730	16
wue11*	female	53	11	1300	55	4	460	9

Demographic and clinical information. Before surgery participants were tested after overnight withdrawal of all dopaminergic medications (meds-off). To evaluate the effect of levodopa (meds-on), the patient had turned into a good quality “on-state” upon receiving 1–1.5-times the levodopa-equivalent of the preoperative morning dose. After surgery, all patients were evaluated in meds-off condition but under chronically effective STN stimulation (meds-off, stim-on). *Indicates the four patients (i.e., wue2, 5, 9 and 11) who were able to complete the motor task (i.e., with both hands in the required amount of time, please refer to *“Task and Experimental Design”* in the “Materials and Methods Section”). DBS, deep brain stimulation; LEDD, levodopa equivalent daily dose; STN, subthalamic nucleus; UPDRS-III, Unified Parkinson Disease Rating Scale motor part.

Demographic and clinical information for all subjects is listed in Table 1. At the time of this study, all patients were on stable dopaminergic treatment (for at least 2 months) and chronically stimulated for 4 months (at least 1 month with unchanged DBS stimulation parameters). The local institutional review board of the University Hospital Wuerzburg approved the study and all patients gave written informed consent.

**Table 1B:** **Molecular imaging data**.

Subject	Percentage loss of DAT binding						STN−	AI Striatum
	Putamen right	Putamen left	Caudate n. right	Caudate n. left	Striatum right	Striatum left		
wue2*	57.87	73.61	43.48	65.22	47.53	67.71	L	47.62
wue3	87.04	85.65	87.75	80.63	86.55	82.06	R	28.57
wue5*	-	-	-	-	-	-	R^§^	-
wue6	57.87	72.69	38.34	48.22	46.19	57.40	L	23.26
wue7	63.43	70.37	51.78	63.64	55.16	65.92	L	27.27
wue9*	82.87	77.78	75.49	71.54	78.03	72.65	R	21.82
wue11*	65.74	63.43	44.27	54.55	52.91	56.95	L	8.96

Percentage loss of DAT binding values calculated with respect to a group of 15 healthy subjects (see Table S1). The DAT binding values of the striatum were used to identify the relative STN− and MC− or STN+ and MC+. We used the whole striatum, rather than its motor part (i.e., the putamen), as the boundaries between the putamen and the caudate nucleus are uncertain in SPECT images. One subject (i.e., wue5) was not willing to perform a SPECT, and STN− or MC− and STN+ or MC+ were based on UPDRS-III score as indicated by §. The clinically most affected hand (higher UPDRS scores) always corresponded to the striatum with less nigro-striatal dopaminergic innervation. The AI was calculated as the relative change of BP_ND_ (see Table S1): AI = ((BP_ND_ striatum ipsilateral − BP_ND_ striatum contralateral)/(BP_ND_ striatum ipsilateral + BP_ND_ striatum contralateral)) x 200. In this case, contralateral refers to the side opposite to the clinically most affected hemibody. For healthy subjects, we considered the average striatal binding of left and right, which did not significantly differ in all subjects. AI, asymmetry index; DAT, dopamine reuptake transporter; BP_ND_, non-displaceable binding potential; L, left; MC, motor cortex; R, right; SPECT, single-photon computed tomography; STN, subthalamic nucleus; UPDRS-III, Unified Parkinson Disease Rating Scale motor part. *Indicates the four patients (i.e., wue2, 5, 9 and 11) who were able to complete the motor task (i.e., with both hands in the required amount of time, please refer to “Task and Experimental Design” in the “Materials and Methods Section”).

### SPECT Data Acquisition and Reconstruction

SPECT data acquisition, reconstruction (Lapa et al., 2015) and analysis has been described in detail previously (Isaias et al., 2010, 2011). All patients but one (i.e., wue5) were willing to perform a SPECT with FP-CIT to measure dopamine reuptake transporter (DAT) density. SPECTs were performed within 3 months before surgery. Scans were started 180 min after injection of 182.3 ± 3.6 MBq of FP-CIT on a dual-headed integrated SPECT/CT system (Symbia T2; Siemens, Erlangen, Germany) in the meds-on condition. In brief, SPECT data were spatially normalized onto a FP-CIT MNI-based template and volumes of interest (VOI) of caudate nucleus, putamen and striatum (for both hemispheres), as well as a reference region in the occipital cortex, were defined using the automated anatomical labeling (Tzourio-Mazoyer et al., 2002). The non-displaceable binding potential (BP_ND_) was then assessed using average regional uptake values from VOI analysis and the occipital cortex as the reference region (Innis et al., 2007). The asymmetry index (AI, expressed as a percent) of whole striatal DAT availability was calculated as the BP_ND_ difference (Striatum_ipsilateral_−Striatum_contralateral_) relative to the mean value of both striatum.

Striatal DAT binding measurements for each patient were compared with normal values of 15 healthy subjects (4 males, 11 females, age range: 44–68 years; Table S1, Supplementary Material). The striatal dopaminergic loss exceeded 50% bilaterally in all but one subject (i.e., wue2, left striatum: 67.7% and right striatum: 47.5%). As previously reported (Panzacchi et al., 2008), the clinically most affected hand (higher UPDRS-III scores) always corresponded to the striatum with less nigro-striatal dopaminergic innervation. The percentage loss of DAT binding values is listed in Table 1 section B. The most affected side was the right one in two out of the four patients who completed the whole study protocol. Based on molecular imaging and clinical data, we identified the hemisphere with less (STN− and motor cortex, MC−) or more (STN+ and MC+) dopaminergic innervation.

**Table 1C:** **Clinical data**.

		wue2	wue5	wue9	wue11
UPDRS-III meds-off	Total hemibody-score right	20	13	12	22
	Total hemibody-score left	10	19	22	13
	Tremor subscore right	4	0	0	0
	Tremor subscore left	0	1	1	0
	Rigidity-bradykinesia subscore right	16	13	12	22
	Rigidity-bradykinesia subscore left	10	18	21	13
UPDRS-III meds-on	Total hemibody-score right	13	10	0	1
	Total hemibody-score left	4	9	7	0
	Tremor subscore right	3	0	0	0
	Tremor subscore left	0	0	0	0
	Rigidity-bradykinesia subscore right	10	10	0	1
	Rigidity-bradykinesia subscore left	4	9	7	0

*UPDRS-III subscores of the four subjects who completed the study protocol. We found a strong lateralization of clinical symptoms. In meds-off condition, the median UPDRS-III score of the most and least affected hemibodies were 22 (range 19–22) and 12 (range 10–13) respectively. UPDRS-III, Unified Parkinson Disease Rating Scale motor part*.

### Task and Experimental Design

The motor tasks have been extensively described in previous studies (Ghilardi et al., 2000; Perfetti et al., 2010; Isaias et al., 2011; Moisello et al., 2015). Briefly, subjects performed a single, multi-joint, uncorrected movement, as accurate and as fast as possible (Figure S1, Supplementary Material). They moved a cursor with either their dominant (right) or non-dominant (left) hand on a digitizing tablet, straight out-and-back, from a central starting point to one of eight equidistant (4 cm) radially-arranged targets that appeared on a screen. An opaque panel prevented the arm vision. All targets were displayed on a screen as circles (2 cm diameter). Targets were presented in random order every 3 s in three blocks of 16 movements each. Participants were tested in meds-off/stim-off condition (i.e., after overnight withdrawal of all dopaminergic drugs and after pausing DBS for at least 1 h) and were asked to perform the task first with the dominant (right) hand and then with the non-dominant (left) hand, irrespective of the more affected body side.

A neurologist (IUI) supervised the absence of any mirror movement or tremor in the hand not performing the task. The data presented refer only to the four subjects who completed the whole study protocol. Three patients were not able to complete the task in the required amount of time (i.e., 3 s per movement) with the right or left hand, and therefore their data were excluded from the analyses.

### Data Recordings and Analysis

For each movement, we measured: onset time (OT, time from target appearance to movement onset), movement time (MT, time from movement onset to reversal), peak velocity (PV) and peak acceleration (PA) and path length (PL, from onset to reversal).

LFPs were recorded with a single bipolar contact configuration for each STN and amplified by 1000. The recording contacts were chosen according to the chronic stimulation setting as a bipolar montage of the two contacts surrounding the stimulation cathode (Devos et al., 2006; Quinn et al., 2015). High density EEG (hdEEG) signals were acquired with a 128-channel EEG Brainamp system (BrainAmp ExG, Brain Product) with sampling frequency at 1000 Hz. LFP and hdEEG recordings were synchronized by means of a common external signal (Figure S2, Supplementary Material), re-sampled at 250 Hz, bandpass filtered in the range 0.5–80 Hz and segmented into 6 s epochs based on the movement onset latencies from −4 s to 2 s after the return-time (RT). RT equals the time point in which the subject came back to the central target (Figure S1, Supplementary Material).

The hdEEG channels affected by bad scalp-electrode were visually identified and replaced with spherical spline interpolation. Trials with sporadic artifacts were excluded by visual inspection. Stereotypical artifacts (e.g., blinks, heartbeat, and muscle tension) were removed by independent component analysis (ICA; Jung et al., 2000; Onton and Makeig, 2006; Onton et al., 2006). For LFPs artifacts management, please refer to the supplementary material (Figure S3). LFP and hdEEG signals were processed and analyzed by means of MatLab-based custom script, EEGLAB (Delorme and Makeig, 2004), Brainstorm (Tadel et al., 2011) and SPM M/EEG Toolbox (Litvak et al., 2011b).

We then calculated the event-related power relative changes (ERD and ERS), normalizing the mean beta power by subtracting and dividing the average power of the whole task interval (from −3 s to 0 s) relative to the RT, multiplied by 100. For efficient spectral estimation of the relatively small number of trials, we used multitaper spectral analysis (Thomson, 1982). We estimated the spectra between −4 s and 2 s relative to the RT of each trial, in overlapping windows of 400 ms with a time resolution of 50 ms. The time-frequency bandwidth was set to 1.5, resulting in two tapers being used. The time-frequency images were then averaged using robust averaging (Wager et al., 2005; Holland and Welsch, 2007; Litvak et al., 2012) and the event-related power changes were obtained by normalizing to the whole trial (−3 s to 0 s) before RT. A representative time-frequency plot showing the cortical and subcortical ERD and ERS is shown in Figure S4 (Supplementary Materials, Methods).

To study cortical beta variations, we assessed the *rebound* in the beta band (13–35 Hz). The *beta-rebound* is the largest peak-to-peak difference between the minimum of the ERD during movement and the maximum of the post-movement ERS in beta band calculated, respectively, between −2 s and −1 s, and between 0 s and 1 s after RT.

Topological maps of these parameters showed two main clusters around CCP3h and CCP4h, as described also in previous studies (Alegre et al., 2005; Meziane et al., 2015; Moisello et al., 2015). Accordingly, we defined two region of interest (ROI) of eight electrodes surrounding CCP3h and CCP4h respectively. The selected electrodes predominantly represent activity over the primary sensorimotor cortex, being the supplementary motor area more medially and the premotor cortex more frontally located (Lalo et al., 2008).

To study the functional connectivity between cortical and subcortical structures, we then computed the coherency between the LFP signals of both the STN and the aforementioned cortical areas (Friston, 2011). We adopted the same method used for spectral estimation for the estimation of coherency. In this case, we also performed a robust averaging (Litvak et al., 2012). Finally, we computed the absolute value of the coherency (i.e., the coherence Coh) and the imaginary part of the coherency (iCoh) to isolate the part of coherency possibly affected by volume conduction (Nolte et al., 2004, 2008; Hohlefeld et al., 2013).

### General Statistical Analysis

Statistical significance of the behavioral performances was assessed by means of a two sample unpaired *t*-Test with a significance level *p* < 0.05.

Beta ERD and ERS were calculated computing the mean beta event-related power changes and then the mean beta *rebound* values for each subject. We assessed significant differences for all the six possible comparisons: −_CONTRA_ vs. +_CONTRA;_ −_IPSI_ vs. +_IPSI;_ −_CONTRA_ vs. +_IPSI;_ −_IPSI_ vs. +_CONTRA;_ −_IPSI_ vs. −_CONTRA;_ +_IPSI_ vs. +_CONTRA_. We used a permutation test for each time point of the beta event-related power changes (with Bonferroni correction for multiple comparisons) and for the beta rebound values. For each comparison (e.g., STN−_CONTRA_ vs. STN+_CONTRA_), we computed the observed statistic (T_obs_) as the difference between the event-related power changes or between the *rebound*, respectively. For constructing the surrogates (T_surr_), we shuffled the trials of each of above listed comparisons (separately), we then recomputed the surrogated event-related power changes and the surrogated beta rebound values. We performed the shuffling 10,000 times with replacement obtaining 10,000 T_surr_ values for each test. The *p* value was computed as the Pr{T_surr_ > T_obs_}. The significance level was set at *p* < 0.05.

Significant regions of Coh and iCoh were determined by statistical comparison to a population of 50 surrogate Coh maps in which any coherence was destroyed. For each pair of channels, the surrogates Coh were generated shuffling the order of trials in one of the two channels. The significance level was set at *p* < 0.05. Multiple comparisons were corrected with false discovery rate method (Benjamini and Yekutieli, 2001). All the analyses were performed in Matlab.

## Results

### Behavioral Data

Movement of the most-affected hand showed a longer MT and lower PV and PA with respect to the less affected hand, though not statistically significant (Table 2). These differences were also present when comparing the movement of the non-dominant vs. dominant hand (Table 2).

**Table 2:** **Behavioral data**.

	Least-affected	Most-affected	Dominant (right)	Non-dominant (left)
OT (ms)	460 (304–693)*	412 (281–600)*	437 (300–675)	435 (289–626)
MT (ms)	725 (378–1092)	766 (425–1273)	699 (383–1074)^•^	792 (413–1278)^•^
PV (cm/s)	8.13 (4.25–15.11)	7.79 (3.76–14.93)	8.34 (4.47–15.01)^**Δ**^	7.58 (3.74–15.06)^**Δ**^
PA (cm/s^2^)	104.09 (32.94–265.65)	103.59 (30.98–275.01)	110.03 (32.52–287.88)^§^	97.65 (31.69–251.81)^§^
PL (cm)	4.82 (3.79–5.92)^⋄^	5.08 (3.91–6.75)^⋄^	5.17 (4.12–6.75)^#^	4.73 (3.72–5.77)^#^
RT (s)	2.04 (1.41–2.78)	2.08 (1.48–2.89)	2.03 (1.4–2.77)	2.05 (1.47–2.87)

In all patients, the most-affected side was contralateral to the striatum with less DAT binding values. All subjects were right-handed, as assessed by a modified Edinburgh handedness inventory. DAT, dopamine reuptake transporter, OT, onset time (time from target appearance to movement onset); MT, movement time (time from movement onset to reversal); PV, peak velocity; PA, peak acceleration; PL, path length (from onset to reversal); RT, return time (the time needed to come back to the central target). Values are expressed as median and range. Statistical significance was assessed by means of a two sample unpaired *t*-Test, ^§^*p* < 0.05 and *^,⋄,•,**Δ**,#^*p* < 0.01.

### Subthalamic Nucleus and Cortical Recordings

**Figure 1:**
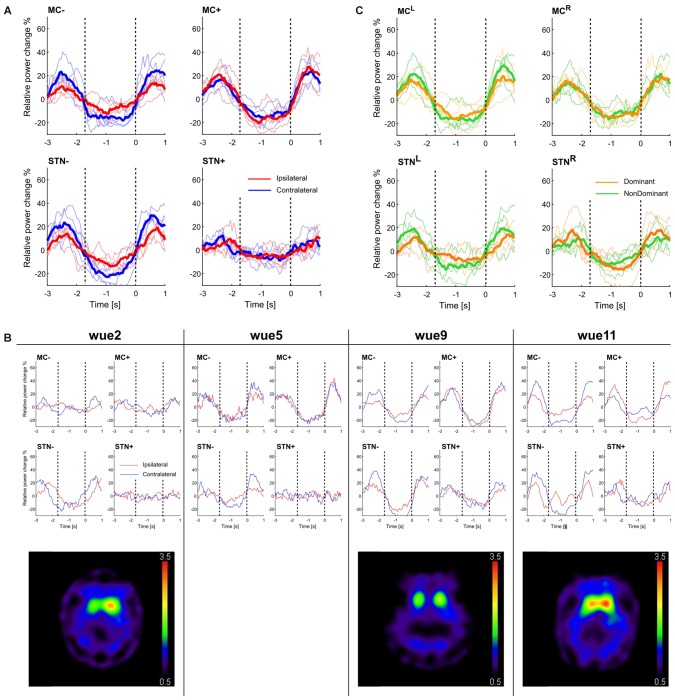
***Beta-rebound* and striatal dopaminergic denervation. (A)** Movement-related beta-modulation with respect to the more and less dopamine-depleted hemisphere. The blue lines represent the movement performed with the hand contralateral to the examined brain structure, red lines with the ipsilateral one. Solid lines represent the average across subjects and thin lines the beta-modulation of each subject. The super-imposed vertical dotted line at 0 s shows the return time (RT). We also indicated with a dotted line at −1.7 s the mean onset time (OT) of all trials (see also Figure S1), as a rough indication of movement OT. **(B)** Movement-related beta-modulation for each subject across all valid trials and the corresponding [^123^I]N-ω-fluoropropyl-2β-carbomethoxy-3β-(4-iodophenyl)nortropane (FP-CIT) and single-photon computed tomography (SPECT) images. **(C)** Movement-related power change in left and right (MC^L^ and MC^R^) and subthalamic nucleus (STN^L^ and STN^R^). The yellow lines represent the movement performed with the dominant hand, the green lines with the non-dominant one. Solid lines represent the average across subjects and thin lines the beta-modulation of each subject. Values are reported in Table 4.

The most striking finding of this study was the stronger β-modulation in the STN of the most dopamine-depleted hemisphere (Figure 1A). Compared to STN+, the STN− (i.e., the one in the hemisphere with less striatal dopaminergic innervation) exhibited greater beta-modulation, both stronger beta-reduction and a higher post-movement rebound, during contralateral hand movements (i.e., the clinically most affected side) and, although weaker, also during ipsilateral hand movements (Tables 3, 4). Beta-modulation in STN+ instead did not significantly differ according to the moving side (Figure 1A; Table 4, subject by subject comparisons are shown in Figure S6A).

**Table 3:** **Beta-oscillation power analyses**.

β-power raw data	wue2		wue5		wue9		wue11	
	IPSI	CONTRA	IPSI	CONTRA	IPSI	CONTRA	IPSI	CONTRA
STN− (mV^2^)	4.48e-02	4.98e-02	3.27e-02	3.46e-02	18.8e-02	18.2e-02	9.08e-02	8.57e-02
STN+ (mV^2^)	1.19e-02	1.17e-02	3.38e-02	3.40e-02	1.82e-02	1.69e-02	2.73e-02	2.50e-02
STN− (-dB)	73.48	73.02	74.85	74.61	67.26	67.40	70.41	70.67
STN+ (-dB)	79.24	79.33	74.71	74.68	77.40	77.71	75.63	76.01

*Spectral power of the neuronal oscillations in the beta frequency range (13–35 Hz) was computed in each subject who completed the study protocol for both the STN and task (i.e., movement with the right and left hand). “IPSI” and “CONTRA” refer to movement performed with the hand ipsilateral or contralateral to the examined STN (STN− or STN+). “−” (MC− and STN−) and “+” (MC+ and STN+) refer instead to the side with less and more striatal dopaminergic innervation or the more and less clinically affected hemibody (for wue05). MC, motor cortex; STN, subthalamic nucleus*.

**Table 4:** ***Beta-rebound* measurements**.

A	STN−CONTRA	STN+CONTRA	STN−IPSI	STN+IPSI	STN−CONTRA	STN+IPSI	STN−IPSI	STN+CONTRA	STN−IPSI	STN−CONTRA	STN+IPSI	STN+CONTRA
wue02	49.94	17.62*	43.95	17.33*	49.94	17.33*	43.95	17.62*	43.95	49.94	17.33	17.62
wue05	51.74	16.90*	25.40	19.19	51.74	19.19*	25.40	16.90	25.40	51.74*	19.19	16.90
wue09	64.74	36.20*	48.51	29.74*	64.74	29.74*	48.51	36.20*	48.51	36.20*	29.74	36.20
wue11	63.93	31.75*	37.01	38.57	63.93	38.57*	37.01	31.75	37.01	63.93*	38.57	31.75

**B**	**MC−CONTRA**	**MC+CONTRA**	**MC−IPSI**	**MC+IPSI**	**MC−CONTRA**	**MC+IPSI**	**MC−IPSI**	**MC+CONTRA**	**MC−IPSI**	**MC−CONTRA**	**MC+IPSI**	**MC+CONTRA**

wue02	32.45	26.84	19.67	22.64	32.45	22.64	19.67	26.84	19.67	32.45*	22.64	26.84
wue05	55.88	57.13	42.54	63.77*	55.88	63.77	42.54	57.13	42.54	55.88	63.77	57.13
wue09	56.76	59.31	31.75	57.39*	56.76	57.39	31.75	59.31*	31.75	56.76*	57.39	59.31
wue11	60.57	34.75*	36.03	63.80*	60.57	63.80	36.03	34.75	36.03	60.57*	63.80	34.75*

Significant differences considering separately the STN (A) and the MC (B) for all the six possible comparisons. Brain structures are distinguished with regards to striatal dopaminergic innervation loss and clinical severity. In each cell, we show the average rebound value, expressed as percentage of relative power change (see also Figure 1 and Figure S6, Supplementary Material, Results). As in Table 3; “IPSI” and “CONTRA” refer to movement performed with the hand ipsilateral or contralateral to the examined STN (STN− or STN+). “−” (MC− and STN−) and “+” (MC+ and STN+) refer instead to the side with less and more striatal dopaminergic innervation or the more and less clinically affected hemibody (for wue05). MC, motor cortex; STN, subthalamic nucleus. *Indicates significance at *p* < 0.05 between each comparison (e.g., STN−_CONTRA_ vs. STN+_CONTRA_).

At a cortical level, MC+ and MC− showed a similar temporal evolution of beta-power, with a steep beta reduction followed by an increase after movement end (Figure 1A). Similar to STN−, the MC− exhibited a more pronounced ERD and ERS for contralateral movements compared to ipsilateral ones. Such a difference was not found in MC+, where movements of both hands evoked similar responses (Table 4, subject by subject comparisons are shown in Figure S6B).

Of note, in one patient with relatively preserved right striatal dopaminergic innervation (<50% loss; i.e., wue2), we showed the smallest beta modulation in the corresponding cortical and subcortical areas (i.e., MC+ and STN+), whereas the patient with the greatest striatal dopaminergic innervation loss ( >70%, bilaterally; i.e., wue9) showed the strongest bilateral beta modulation (Figure 1B).

When grouping the data by handedness, we found a similar time evolution of beta-power between the two MC (i.e., MC^L^ and MC^R^) and of the two STN (i.e., STN^L^ and STN^R^), regardless of the moving hand (Figure 1C).

**Figure 2:**
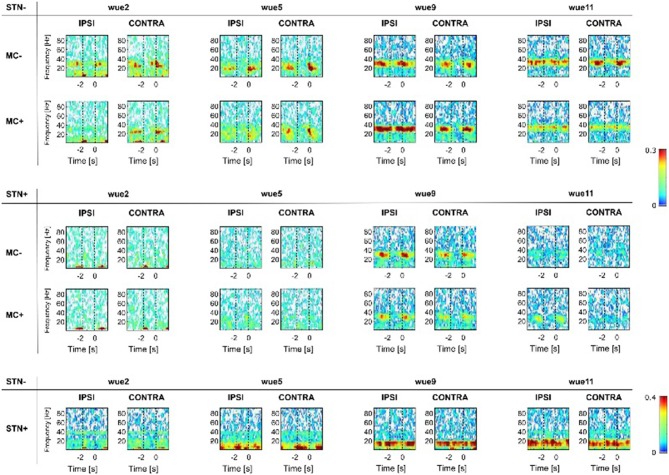
**Coherence analyses.** Subcortical- (i.e., ^Coh^STN−/STN+) and cortical-subcortical coherence (i.e., ^Coh^STN−/MC−, ^Coh^STN−/MC+ and ^Coh^STN+/MC−, ^Coh^STN+/MC+) are reported for each subject with respect to STN− and STN+. White color shows lack of coherence. From blue to red color we show increasing significant coherence between brain structures. Results were mirrored by iCoh, thus supporting the lack of volume conduction artifact (Supplementary Material, Results, Figure S8). As in Figure 1, the super-imposed vertical dotted line at 0 s shows the RT. We also indicated, with a dotted line at −1.7 s, the mean OT of all trials (see also Figure S1) as a rough indication of movement OT. “IPSI” and “CONTRA” refer to movement performed with the hand ipsilateral or contralateral to the examined STN (STN− or STN+). “−” (MC− and STN−) and “+” (MC+ and STN+) refer instead to the side with less and more striatal dopaminergic innervation or the more and less clinically affected hemibody (for wue05). MC, motor cortex; STN, subthalamic nucleus. MC, motor cortex; STN, subthalamic nucleus.

Cortical-subcortical (i.e., ^Coh^MC−/STN−, ^Coh^MC+/STN− and ^Coh^MC−/STN+, ^Coh^MC+/STN+) and subcortical (i.e., ^Coh^STN−/STN+) coherences are shown in Figure 2. Each patient displayed a distinctive frequency of coherence within the beta range. Cortical-subcortical coherencies diminished during movement execution (i.e., from −2 s to 0 s). In all patients, ^Coh^MC−/STN− was greater than ^Coh^MC+/STN− and ^Coh^MC+/STN+ irrespective of the moving hand. Of note, the patient with the greatest striatal DAT loss (i.e., wue9) showed the most persisting and strongest cortical-subcortical coherences, also in the MC+ hemisphere (Figure 2, Supplementary Figures S7,S8).

We also found a significant subcortical, cross-hemispheric coupling (i.e., ^Coh^STN−/STN+), although weaker than the ipsilateral cortical-subcortical ones. This subcortical coherence was not affected by movement (Figure 2) and mirrored the cortical-subcortical coherence, being higher in the patient with the greatest bilateral striatal DAT loss (i.e., wue9; Figure 2, Supplementary Figures S7, S8).

## Discussion

Our findings suggest that movement-related beta-modulation is dependent on striatal dopaminergic innervation. Specifically, we described greater modulation of STN activity in the hemisphere with less dopaminergic innervation in three subjects with PD and STN-DBS, in particular for movements performed with contralateral hand (Figure 1).

These data provide preliminary evidence of a role of striatal dopamine for precise cortical-subcortical tuning of movement, and distinctive cortical-basal ganglia motor processing of ipsi- and contralateral movements (Devos et al., 2006). In line with our findings, several studies reported excessive beta oscillations in the STN of PD patients, which was reduced by voluntary movements (for review, Hammond et al., 2007; Brittain and Brown, 2014). One study, with self-initiated left and right wrist extensions in alternating series, also showed greater STN beta-modulation in the most affected body side of PD patients (Alegre et al., 2005).

Coherency analyses, a measurement of functional connectivity (Friston, 2011), can serve to study the cortical-basal ganglia network organization. Indeed, the functional segregation among cortical-basal ganglia loops might rely on distinct anatomical connections, but also be frequency-dependent through the coupling of precise activities at specific frequency bands (Fogelson et al., 2006; Lalo et al., 2008). We can further assume that cortical-subcortical (beta-) coherency reflects the number of coupled neurons (Marsden et al., 2001; Cassidy et al., 2002; Lalo et al., 2008). Most of the studies addressing coherency of cortical-basal ganglia circuitry were performed at rest (Williams et al., 2002; Fogelson et al., 2006; Hirschmann et al., 2011; Litvak et al., 2011a; Kato et al., 2015). These studies described an excessive subcortical- and cortical-subcortical coupling in subjects with PD in meds-off state (Williams et al., 2002; Fogelson et al., 2006; Hirschmann et al., 2011; Litvak et al., 2011a; Kato et al., 2015). Two studies also investigated the effect of levodopa and voluntary movements, but with inconsistent results (Lalo et al., 2008; Hirschmann et al., 2013). In particular, Lalo et al. (2008) described a movement-related drop of cortical-subcortical coupled beta-activity, possibly driven by the cortex, during a repetitive hand flexion-extension task. In this study, coherencies were not influenced by levodopa. On the contrary, Hirschmann et al. (2013) reported a significant reduction of cortical-subcortical coupling after levodopa intake during the execution of a simple motor task, that is opening-closing hand. In all but one patient (i.e., wue11) in our study, cortical-subcortical coherence was greatly diminished during movements and reappeared in rest intervals, predominantly in the hemisphere with less striatal dopaminergic innervation (i.e., STN− and MC−; Figure 2). Anecdotally, the patient with the strongest cortical-subcortical coherence also in the less affected hemisphere (i.e., ^Coh^MC+/STN+; i.e., wue9) showed the greatest bilateral loss of striatal dopamine (also in the less affected striatum [left side]: 72.65%, Table 1). In line, ^Coh^MC+/STN+ were absent in the two patients with overall higher DAT bindings values (i.e., wue2 and wue11; Table 1). Taken together, these data suggest a direct influence of striatal dopamine on cortical-subcortical coherencies during movement and support a role for striatal dopamine in uncoupling cortical and subcortical networks.

Finally, we also measured subcortical cross hemispheric coupling during movement (i.e., ^Coh^STN−/STN+, Figure 2). In line with previous measurements at rest (de Solages et al., 2010; Kato et al., 2015), we showed a subject-specific Coh in the beta-range between the two STNs (Figure 2). Of relevance, such subcortical cross hemispheric coherence was not modulated by movements, despite the differences in beta-power between STN+ and STN− (Figure 1A), and it did not mirror the movement-related drop of the cortical-subcortical coherence (Figure 2). Our findings are consistent with recent studies, though with different tasks, showing a lack of modulation of subcortical cross-hemispheric coupling in the beta-band during movements in subjects with PD (Darvas and Hebb, 2014; Kato et al., 2016). We speculate that such a persistent subcortical coherence might not be related to motor processing but relies upon a (bilateral) dopaminergic loss.

Our study has several limitations, in particular the small sample size, although in the range of previous reports (Cassidy et al., 2002; Priori et al., 2002; Alegre et al., 2005). The exiguous number of patients able to complete the study protocol did not allow defining whether the role of striatal dopamine deteriorates linearly or step-wise along with disease progression. Furthermore, we were not able to disentangle the effect of an unbalanced dopaminergic activity between the two hemispheres. It is worth noting that all patients showed a bilateral dopaminergic loss (Table 1). Besides the extent of dopaminergic striatal innervation *per se*, it is tempting to speculate that the asymmetry of this denervation might also play a role in the cortical-subcortical processing of motor commands.

Another limitation of this study is the focus on beta band modulation. This choice was based on available data suggesting that excessive beta-activity is either related to or causing bradykinesia in PD (Hammond et al., 2007; Eusebio and Brown, 2009; Brittain and Brown, 2014). Moreover, it was also shown that movement-related cortical-subcortical modulation happens specifically in the beta frequency band (Lalo et al., 2008; see also Figure 1 and Supplementary Figure S5).

Despite these limitations, it is worth mentioning that in this study the LFPs of STN were recorded months after surgery by means of a new, fully implantable device. Delayed recordings decrease the influence of high impedance variability and of microlesioning effect, which influence immediate post-operative recordings (Lalo et al., 2008).

Our conclusions are presumptive, but support the notion of a dopaminergic role in shielding subcortical structures from an excessive cortical entrapment and cross hemispheric coupling, thus allowing fine tuning of movement (Hammond et al., 2007). Furthermore, in patients with PD an unbalanced modulation of motor processing between the two hemispheres, which reflect the degree of dopamine loss and the lateralization of clinical symptoms, might have relevant therapeutic implications. The success of adaptive or patterned stimulation protocols should also take into account dopamine-dependent STN neuronal activity to offer more symptom-targeted stimulation effects than conventional DBS.

## Author Contributions

IUI, MFG, GP, CM and JV conceived and designed the experiments. IUI, AC, NGP, GA, JB, MMR, CM and FS organized and analyzed the raw data. IUI, AC, NGP, GA, JB, MMR and FS participated in the statistical analysis and interpretation of data. AC, NGP, GA, JB, MMR and FS wrote the article, and IUI, MFG, GP, CM and JV revised the manuscript.

## Funding

The study was sponsored in part by the “Interdisziplinäres Zentrum für Klinische Forschung (IZKF)” of the University Hospital Wuerzburg and by the “Fondazione Grigioni per il Morbo di Parkinson”. NGP was supported by a grant of the German Excellence Initiative to the Graduate School of Life Sciences, University of Wuerzburg and IRCCS “C. Mondino”, Pavia.

## Conflict of Interest Statement

The authors declare that the research was conducted in the absence of any commercial or financial relationships that could be construed as a potential conflict of interest.

## Abbreviations

BP_ND_: non-displaceable binding potential
DAT: dopamine reuptake transporter
DBS: deep brain stimulation
ERD: event-related desynchronization
ERS: event-related synchronization
FP-CIT: [^123^I]N-ω-fluoropropyl-2β-carbomethoxy-3β-(4-iodophenyl)nortropane
ICA: independent component analysis
iCoh: imaginary part of the coherency
LEDD: levodopa equivalent daily dose
LFPs: local field potentials
MC: Motor cortices
MT: movement time
OT: onset time
PA: peak acceleration
PL: path length
PV: peak velocity
ROI: region of interest
RT: return time
SPECT: single-photon computed tomography
STN: Subthalamic Nucleus
UPDRS-III: Unified Parkinson Disease Rating Scale motor part
VOI: volume of interest.
