# Supplementary material for: Striatal Dopaminergic Innervation Regulates Subthalamic Beta-Oscillations and Cortical-Subcortical Coupling during Movements: Preliminary Evidence in Subjects with Parkinson’s Disease
Source: Front Hum Neurosci. 2016 Dec 6;10:611. doi: 10.3389/fnhum.2016.00611 (PMC5138226; doi:10.3389/fnhum.2016.00611)
Supplement: Supplementary file 1 [file DataSheet_1.docx]

Supplementary Material

Striatal dopaminergic innervation regulates subthalamic beta-oscillations and cortical-subcortical coupling during movements: evidence in three subjects with Parkinson’s disease

**Andrea Canessa^1^, Nicolò G. Pozzi^1^, Gabriele Arnulfo^1^, Joachim Brumberg^2^, Martin M. Reich^1^, Gianni Pezzoli^4^, Maria F. Ghilardi^5^, Cordula Matthies^3^, Frank Steigerwald^1^, Jens Volkmann^1^ and Ioannis U. Isaias^1^***

1. Department of Neurology, University Hospital and Julius-Maximilian-University, Wuerzburg, DE
2. Department of Nuclear Medicine, University Hospital and Julius-Maximilian-University, Wuerzburg, DE
3. Department of Neurosurgery, University Hospital and Julius-Maximilian-University, Wuerzburg, DE
4. Centro Parkinson ASST G. Pini-CTO, Milan, IT
5. Department of Physiology, Pharmacology and Neuroscience, CUNY Medical School, New York, NY, USA

* Correspondence: Ioannis U. Isaias, Mail: Isaias_I@ukw.de

## SUPPLEMENTARY MATERIAL – METHODS

### Task and experimental design

*
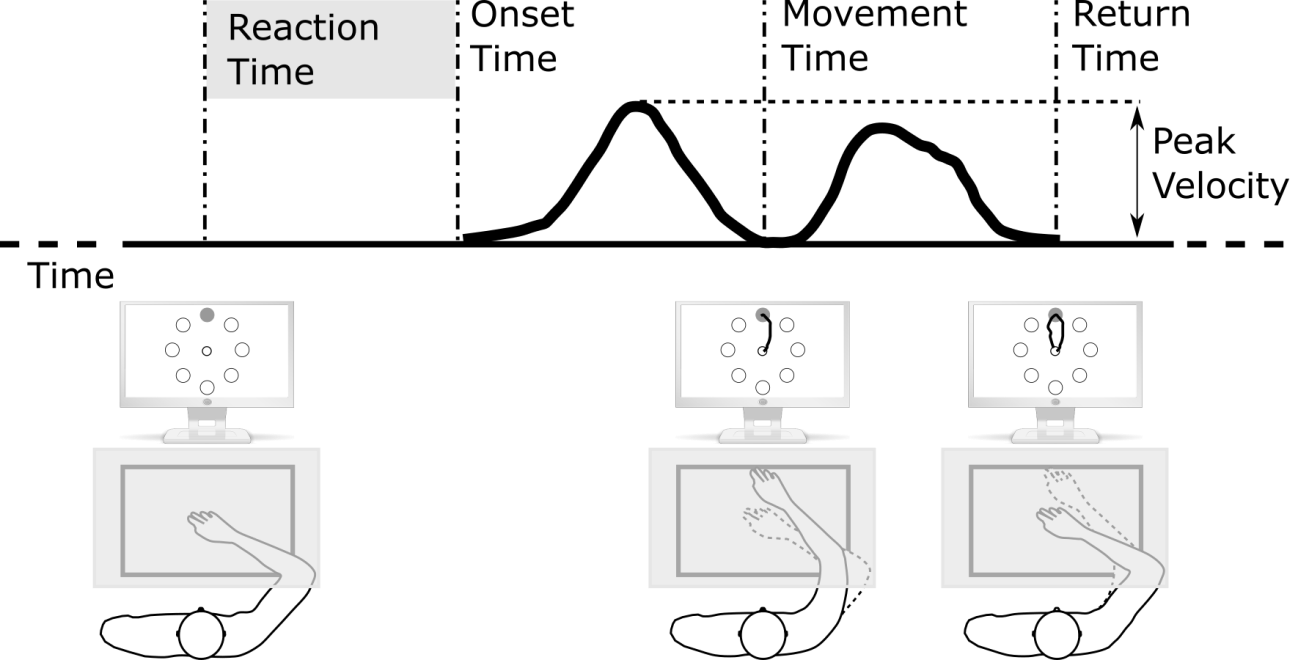
*

***Figure S1.*** *Experimental setup. All subjects moved a cursor on a digitizing tablet and performed out-and-back movements toward one of eight targets presented on the computer screen every 3 s. An opaque panel prevented the arm vision. The black line represents the velocity of the movement reaching the target and coming back to the center.*

**SPECT data acquisition and reconstruction**

| Subject | Caudate n.  right | Putamen  right | Striatum  right | Caudate n.  left | Putamen  left | Striatum  left |
| --- | --- | --- | --- | --- | --- | --- |
| wue2 | 1.43 | 0.91 | 1.17 | 0.88 | 0.57 | 0.72 |
| wue3 | 0.31 | 0.28 | 0.30 | 0.49 | 0.31 | 0.40 |
| wue5 | - | - | - | - | - | - |
| wue6 | 1.56 | 0.91 | 1.20 | 1.31 | 0.59 | 0.95 |
| wue7 | 1.22 | 0.79 | 1.00 | 0.92 | 0.64 | 0.76 |
| wue9 | 0.62 | 0.37 | 0.49 | 0.72 | 0.48 | 0.61 |
| wue11 | 1.41 | 0.74 | 1.05 | 1.15 | 0.79 | 0.96 |
| HC1 | 2.57 | 2.33 | 2.18 | 2.56 | 2.45 | 2.28 |
| HC2 | 3.39 | 3.03 | 3.14 | 3.33 | 2.88 | 3.01 |
| HC3 | 2.13 | 1.83 | 1.91 | 2.11 | 1.89 | 1.90 |
| HC4 | 2.61 | 2.13 | 2.21 | 2.53 | 2.30 | 2.30 |
| HC5 | 2.17 | 1.91 | 1.99 | 2.03 | 2.14 | 2.08 |
| HC6 | 1.65 | 1.43 | 1.47 | 1.43 | 1.57 | 1.44 |
| HC7 | 2.53 | 2.01 | 2.18 | 2.50 | 2.03 | 2.18 |
| HC8 | 2.52 | 1.97 | 2.23 | 2.53 | 2.29 | 2.40 |
| HC9 | 2.59 | 2.36 | 2.22 | 2.76 | 2.32 | 2.30 |
| HC10 | 3.46 | 3.07 | 3.11 | 3.39 | 3.10 | 3.15 |
| HC11 | 3.52 | 3.03 | 3.13 | 3.66 | 2.88 | 3.21 |
| HC12 | 2.27 | 1.93 | 2.00 | 2.19 | 1.97 | 2.00 |
| HC13 | 2.63 | 2.30 | 2.42 | 2.77 | 2.57 | 2.50 |
| HC14 | 2.80 | 2.17 | 2.24 | 2.52 | 2.10 | 2.23 |
| HC15 | 2.40 | 1.98 | 2.01 | 2.22 | 1.97 | 2.00 |
| HC_Mean_ | 2.62 | 2.23 | 2.30 | 2.57 | 2.30 | 2.33 |

***Table S1.*** *Non-displaceable binding potentials (BP_ND_) of the PD patients and of 15 healthy controls (HC). The BP_ND_ for caudate nucleus, putamen and whole striatum (for both hemispheres) was assessed using average regional uptake values from volumes of interest analysis and the occipital cortex as the reference region (Innis et al., 2007).*

### High density EEG and Activa PC+S® synchronization

### In the currently available Activa PC+S® device, an external trigger signal cannot be fed directly to the recording system. Therefore, to synchronize the STN and hdEEG recordings we exploited an electrical artefact simultaneously visible by both the hdEEG sensors and the Activa PC+S® electrodes. This electrical artefact was originated by a Transcutaneous Electrical Nerve Stimulation (TENS) unit with electrodes placed on the hdEEG cap and on the neck, above the internalized Activa PC+S® wire. The electrode on the hdEEG cap was placed directly over the site of the burr hole. The stimulating pulses had voltage amplitude up to 7 V (median: 4 V, range: 3 V-7 V) and an oscillation frequency of 130 Hz (Figure S2A). We gave two TENS, one at the beginning and one at the end of the recording session (Figure S2B). The two TENS allowed us to estimate the correct sampling frequency of the Activa PC+S®. The manufacturer documentation reported that the real sampling frequency could vary around its nominal value of 422 Hz (mean: 421.9409 Hz, standard deviation: 0.1763 Hz). To synchronize the two devices, we (1) identified the time point when the last peak of the two artefactual patterns occurred, both in the Activa PC+S® and in the hdEEG recordings; we then (2) computed the correct sampling frequency of the Activa PC+S®, assuming the hdEEG sampling frequency equal to 1KHz, (3) resampled all the recordings at 400 Hz and (4) aligned the Activa PC+S® and hdEEG recordings (Figure S2C).

**
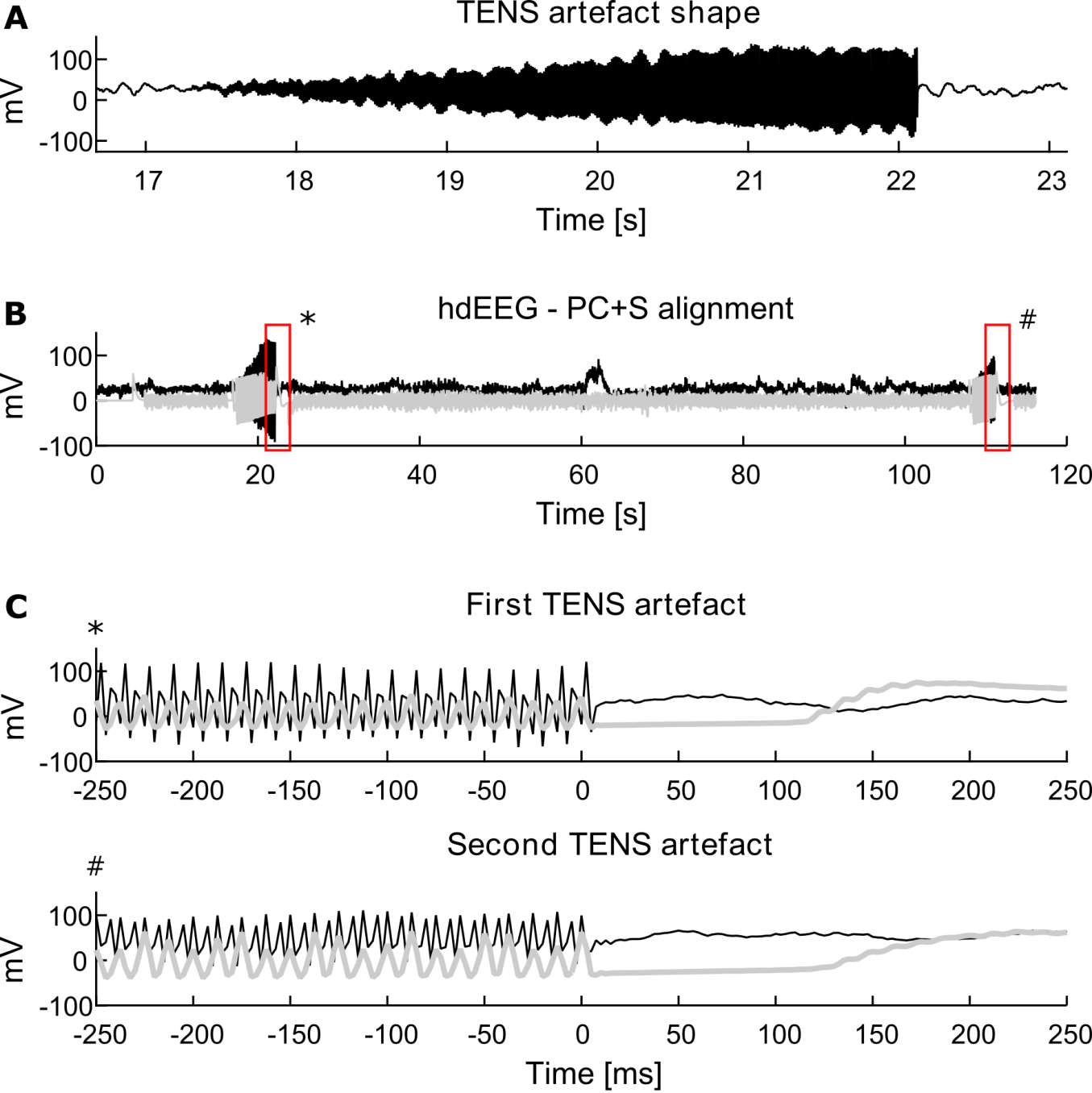
**

***Figure S2. HdEEG and Activa PC+S® synchronization.*** *(A)The shape of the TENS artefact recorded by the hdEEG was characterized by a slow increment up to the maximum value but a very sharp switch off. (B) Example of hdEEG (black line) and Activa PC+S® (grey line) signal alignment. (C) A close-up view of the two TENS artefacts is shown in part B (red boxes, * first TENS and # second TENS).*

#### Artefact of the Activa PC+S® neurostimulation system in the recordings

#### We encountered three artefactual oscillatory components in the recording of Activa PC+S® neurostimulation system (Medtronic Inc., Neuromodulation, Minneapolis, USA).

#### The first artefact was characterized by a sharp high-amplitude biphasic transition of ≈500 ms. This artefact might have occurred when the device performed an interrogation of its internal status. Despite the signal to noise ratio (SNR) of the artefact, it was rarely recorded (i.e. 10 events in ≈20 min of recording) and we rejected the time intervals/trials displaying it (Figure S3A).

#### The second artefact was characterized by a rhythmicity centered at a quarter of the chosen sampling frequency. This was due to the device internal clock and the multiplexer sampling circuitry. In our case, having a sampling frequency of 422 Hz, the artefact was at 105 Hz and therefore outside the band of interest. In any case, it could have been eliminated with a properly designed notch filter.

#### The third and most relevant artefact was the interference from cardiac electrical activity (Figure S3B-D). This was due to a leakage of fluid through the neurostimulator that affected the most ventrally-located channels. The fluid makes a low resistance path for current flow, reducing the common mode rejection ratio (CMRR) of the differential amplifier, and it allows the cardiac artefact to be added to the signal recorded from the implanted electrodes (Quinn et al., 2015). The SNR of this artefact was greatly variable, thus making the ECG interference either clearly visible or hidden within the neural signal. The main problem of this artefact was that its power spectral support extended from very low frequency up to 40 Hz, thus affecting both the alpha and beta frequency bands. The solution adopted to mitigate the cardiac artefact exploits a recorded raw ECG signal. We identified the QRS peaks in the raw ECG signal and we epoched the recorded signals around these events. Once we had the epochs locked around the ECG peaks, we averaged them to obtain a mean artefactual signal to be subtracted from the original signal (Figure S1B-D). We operated this subtraction only when the averaged signal displayed a peak higher than a threshold, which was fixed at three times the standard deviation. The standard deviation was computed in the time intervals not belonging to the artefactual epochs. In our analysis we adopted this solution in two out of eight STN sides (two/four subjects).

**
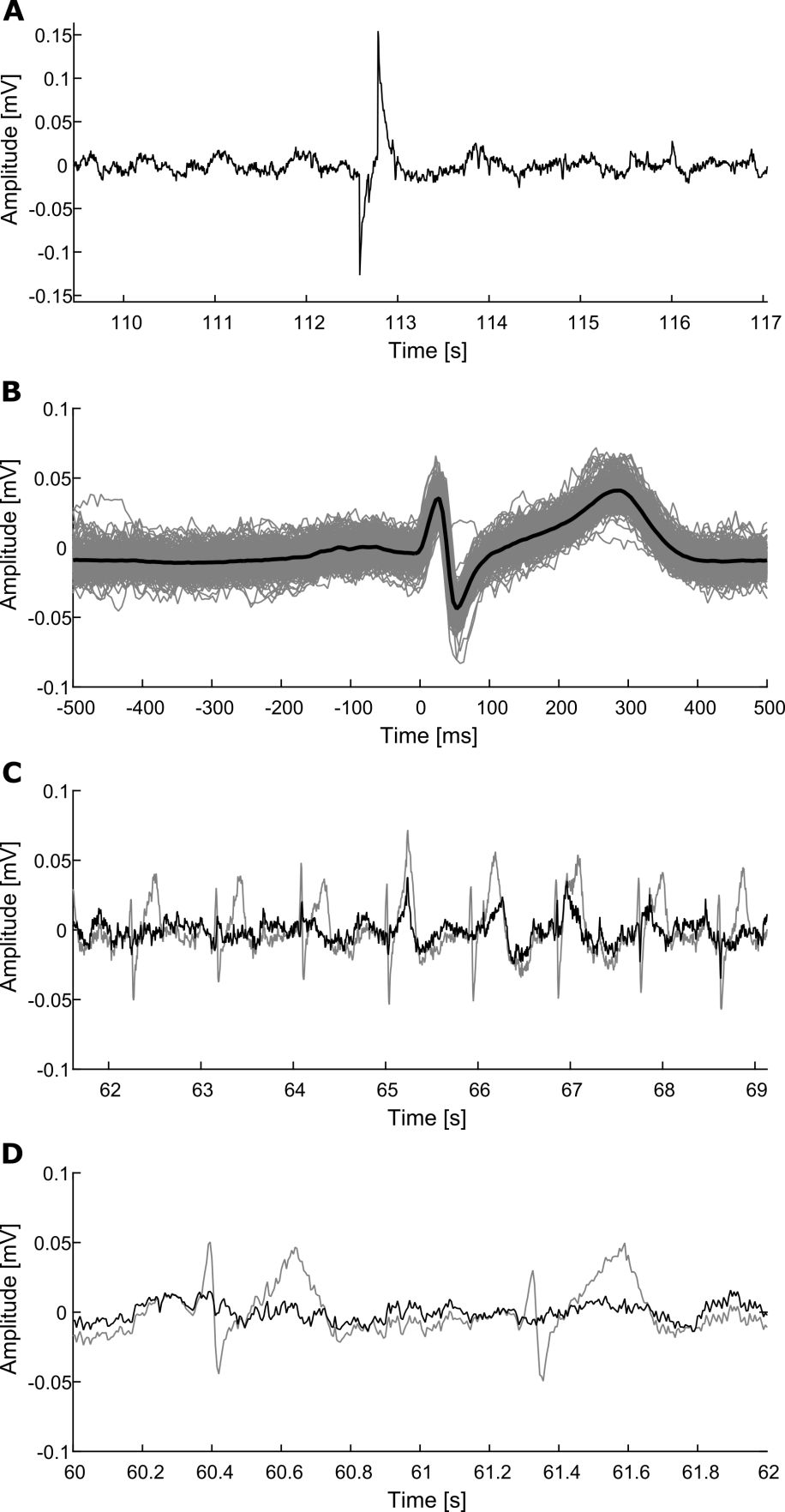
**

***Figure S3.*** *Artefactual components of Activa PC+S® recordings. (A) Biphasic transition due to internal interrogation. (B-C-D). Artefacts from cardiac electrical activity. (B) Gray lines represent all the epochs aligned at the QRS peak, which was identified in the raw ECG signal; the black line displays their mean (i.e. average cardiac artefact). (C) Grey line shows the original signal recorded by the Activa PC+S® while the black line is the pruned signal, obtained subtracting the mean artefact. (D) A close-up view of part C.*

### Time-frequency plot of subthalamic LFPs

### We calculated the event-related power relative changes (ERD and ERS) normalizing the mean beta power by subtracting and dividing the average power of the whole task interval (from -3 s to 0 s) relative to the return-time, multiplied by 100. We used multitaper spectral analysis for efficient spectral estimation and assessed the spectra between -4 s and 2 s relative to the return-time of each trial. Several overlapping windows of 400 ms with a time resolution of 50 ms were used for this analysis. The time-frequency bandwidth was set to 1.5, resulting in two tapers being used. We than averaged the time-frequency images by means of robust averaging and calculated the event-related power changes by normalizing to the whole trial (-3 s to 0 s) before return-time.


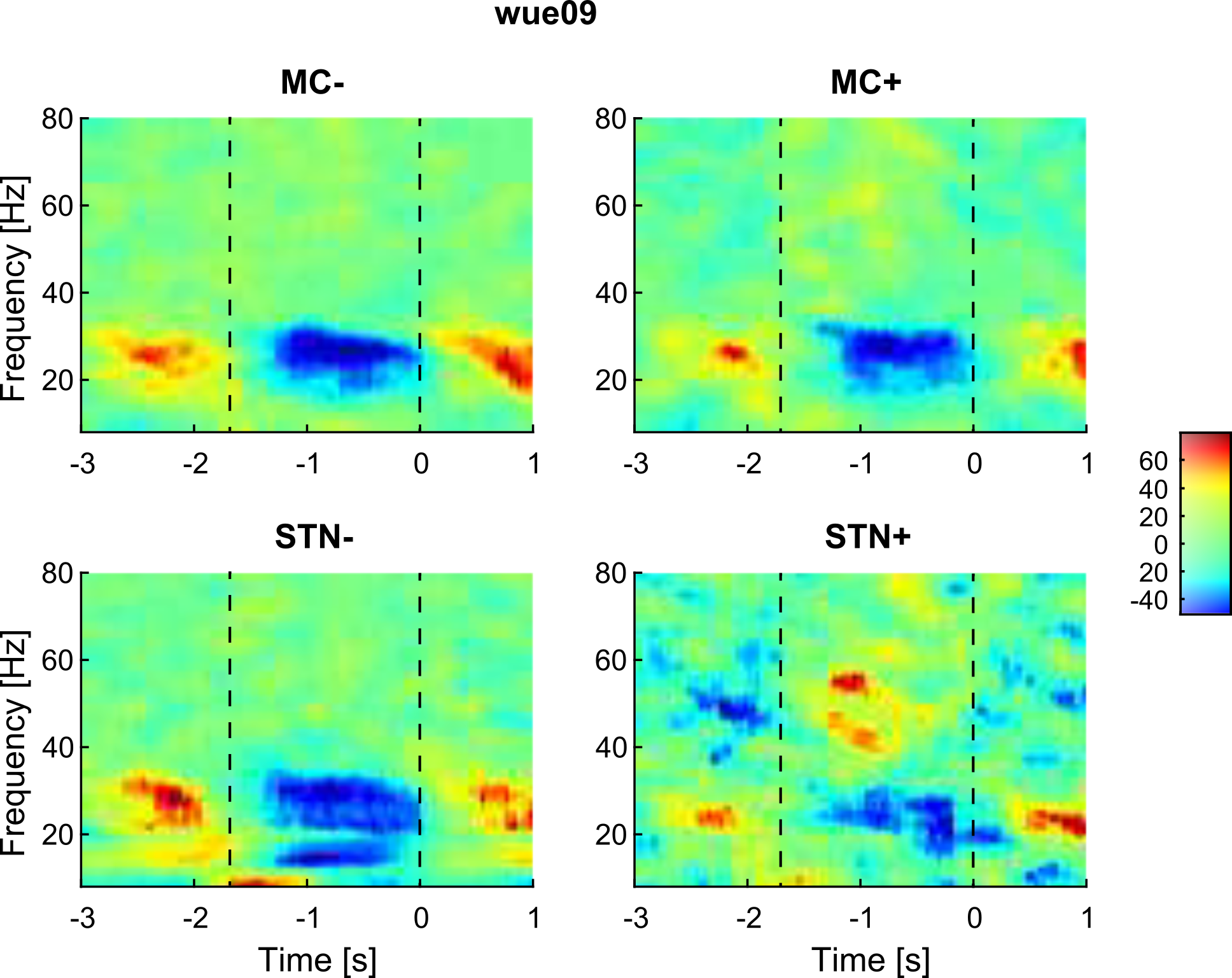


***Figure S4.*** *Time-frequency plot and movement-related ERD/ERS of one subject (wue09). Cortical and subcortical beta-ERD is clearly visible in the time window corresponding to the movement execution (i.e. ≈-2 s to 0), and followed by ERS in the beta-frequency range after movement termination. The super-imposed vertical dotted line at 0 s shows the return time. We also indicated with a dotted line at -1.7 s the mean onset time of all trials (see also Figure S1), as a rough indication of movement onset time.*

**SUPPLEMENTARY MATERIAL – RESULTS**

**Subthalamic nucleus and cortical recordings**

###
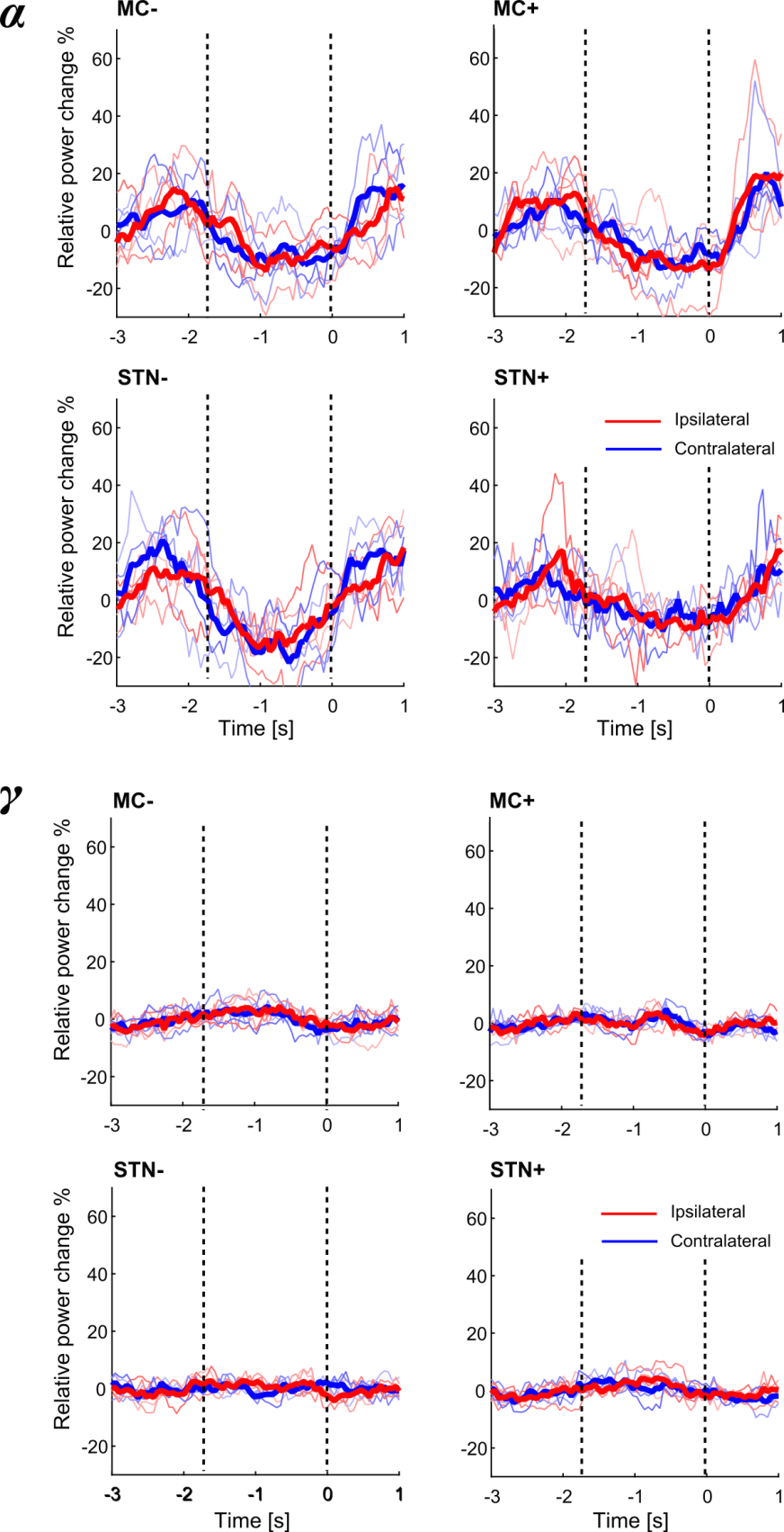


***Figure S5.*** *Alpha and gamma modulation in the motor cortex and in the subthalamic nucleus. The movement-related power change in the alpha (≈7 Hz -12 Hz) and gamma (≈35 Hz -100 Hz) band is displayed with respect to the more- and less-dopamine depleted hemisphere. The blue lines represent the movement performed with the hand contralateral to the examined structure, the red lines with the ipsilateral hand. Solid lines represent the average across subjects and thin lines the beta-modulation of each subject. The super-imposed vertical dotted line at 0 s shows the return time. We also indicated with a dotted line at -1.7 s the mean onset time of all trials (see Figure S1), as a rough indication of movement onset time.*

### Movement-related beta-modulation

### In all subjects, the STN– (i.e. the STN in the hemisphere with less striatal dopaminergic innervation) exhibited greater beta modulation with respect to the STN+ (Figure S6 part A, column I-IV). Stronger beta-reduction and a higher post-movement rebound were also found in the STN– compared to the STN+, for both ipsilateral and contralateral hand movements (Figure S6 part A, column I-IV). With regards to hand movements, beta-modulation in STN– was higher during contralateral than ipsilateral hand movements, this was not found in STN+ (Figure S6 part A, column V, VI).

### At a cortical level, MC+ and MC– showed a similar temporal evolution of beta-power, with a steep beta reduction followed by an increase after movement end (Figure S6 part B). Similar to what reported subcortically the MC– exhibited a more pronounced ERD and ERS for contralateral movements compared to ipsilateral ones (Figure S6 part B, column V). Such a difference was not found in MC+, where movements of both hands evoked similar responses (Figure S6 part B, column VI).


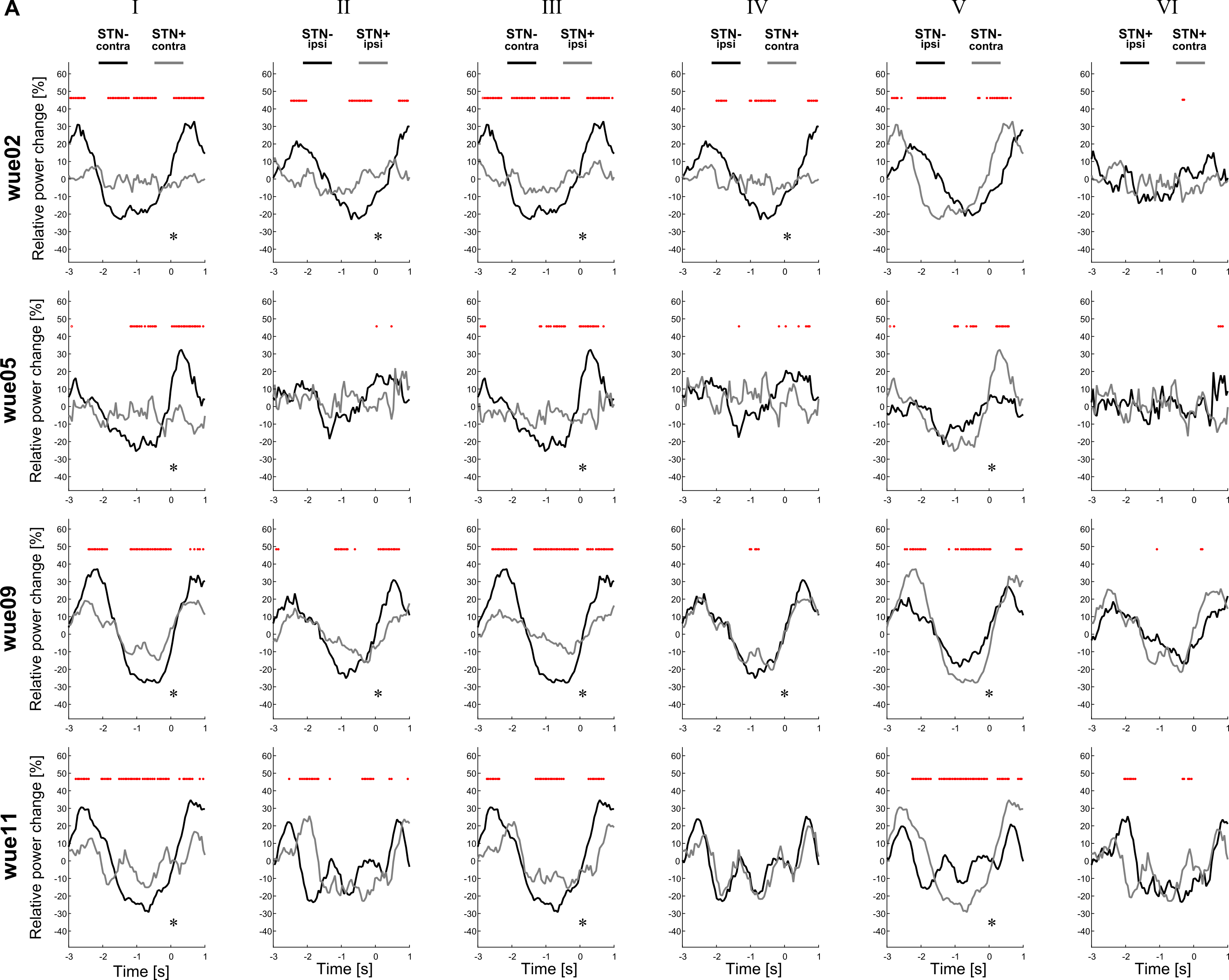

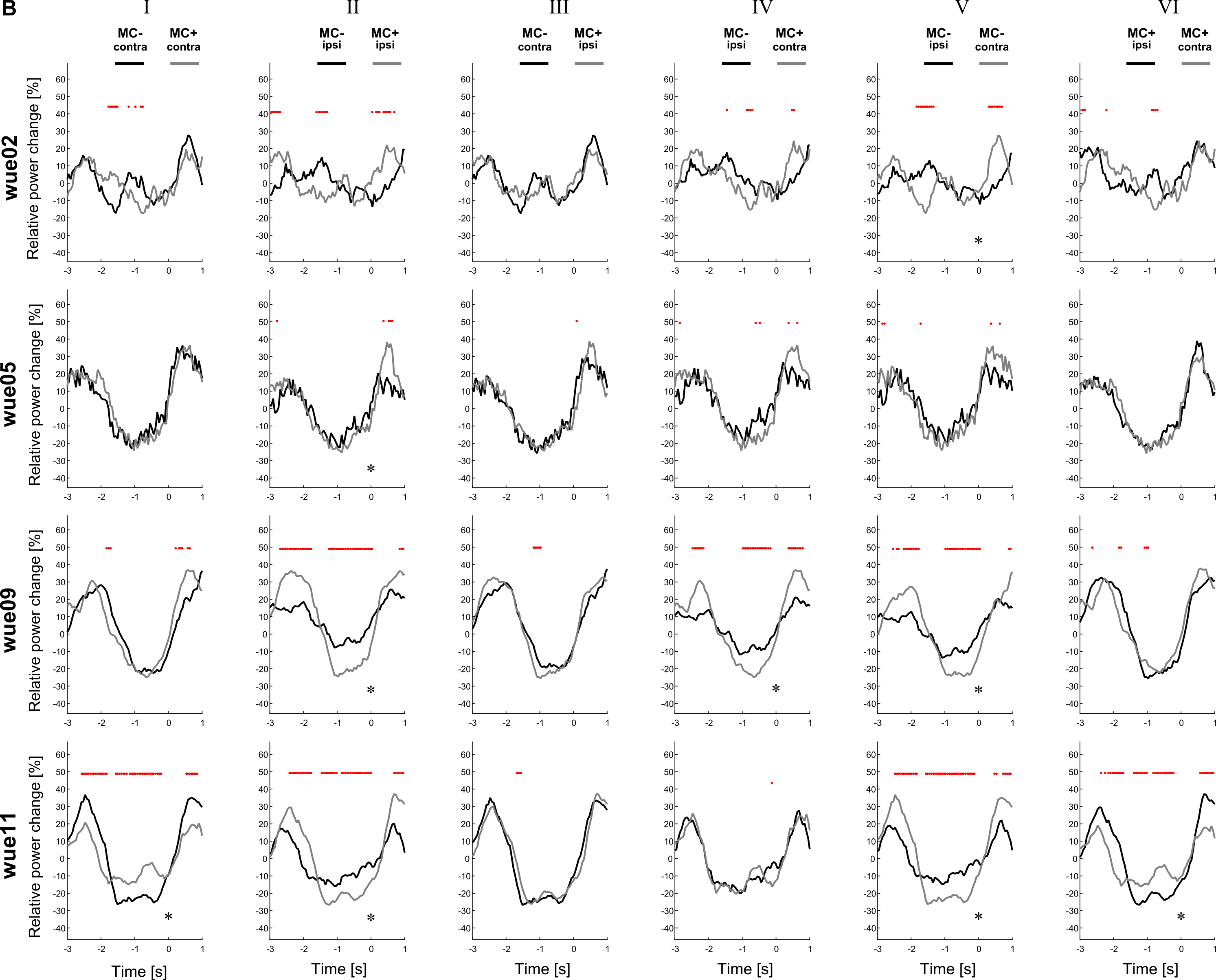


***Figure S6.*** *Cortical and subcortical movement-related beta-modulation. The movement-related power change in the beta band (≈13 Hz -35 Hz) is displayed with respect to the more (STN****–*** *and MC****–****) and less dopamine-depleted (STN+ and MC+) hemisphere for all subject and all the possible movements. We show in (A) subcortical and in (B) cortical movement-related beta-modulation. Subjects’ ID is written on the left of each raw and comparisons on top of each column. The difference for each point of the beta-power during movements was assessed with a permutation test (with Bonferroni correction for multiple comparisons); statistical significance is shown with red dots. Differences of rebound values were also assessed with a permutation test. * indicates significance at p<0.05 between each comparison (e.g. STN–_CONTRA_ vs. STN+_CONTRA_)*

**Coherence analysis**

Figure S7 and S8 provide additional findings on coherence analysis. In particular, we focused more closely on coherence modulation in the beta-frequency (≈15-30Hz) over time in each subject with respect to STN– and STN+ (Figure 7S). As also showed in Figure 2 (Main text), cortical-subcortical coherence decreased during movement execution, whereas subcortical beta-coherence remained more stable.

We also computed (Figure S8) the imaginary part of the coherency (iCoh), to avoid possible artefactual results caused by volume conduction, and all results were confirmed. Indeed, cortical-subcortical (i.e. iCohMC–/STN–, iCohMC+/STN– and iCohMC–/STN+, iCohMC+/STN+) and subcortical (i.e. iCohSTN–/STN+) coherency were patient specific, being present in distinctive frequency in different patients, but all within the beta range (Figure S8). In all patients, iCohMC–/STN– was greater than iCohMC+/STN– and iCohMC+/STN+ irrespective of the moving hand. Cortical-subcortical coherency were reduced during movement execution (i.e. from -2 to 0s). We also found a significant subcortical, cross-hemispheric coupling (i.e. iCohSTN–/STN+). Of interest, this subcortical coherency was not affected by movement (Figure S8).


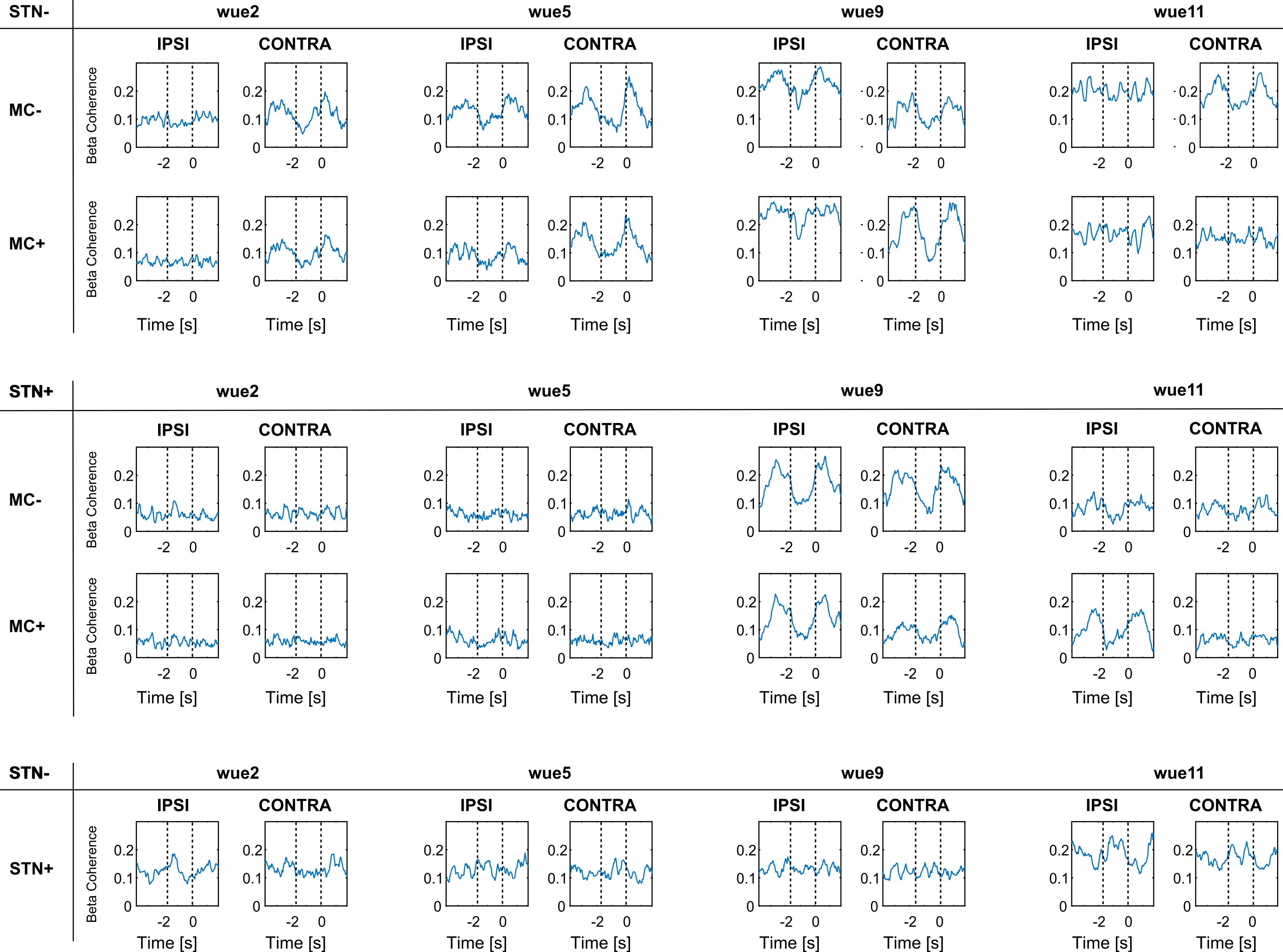


***Figure S7.*** *Subcortical- (i.e. CohSTN–/STN+) and cortical-subcortical (i.e. CohSTN–/MC–, CohSTN–/MC+ and CohSTN+/MC–, CohSTN+/MC+) coherence in the beta-frequency band (≈15-30Hz) of each subject are showed according to STN– and STN+. The super-imposed vertical dotted line at 0 s shows the return time. We also indicated, with a dotted line at -1.7 s, the mean onset time of all trials as a rough indication of movement onset time. “IPSI” and “CONTRA” refer to movement performed with the hand ipsilateral or contralateral to the examined STN (STN– or STN+). “–” (MC– and STN–) and “+” (MC+ and STN+) refer instead to the side with less and more striatal dopaminergic innervation or the more and less clinically affected hemibody (for wue05). MC = motor cortex, STN = subthalamic nucleus. MC = motor cortex, STN = subthalamic nucleus.*

**
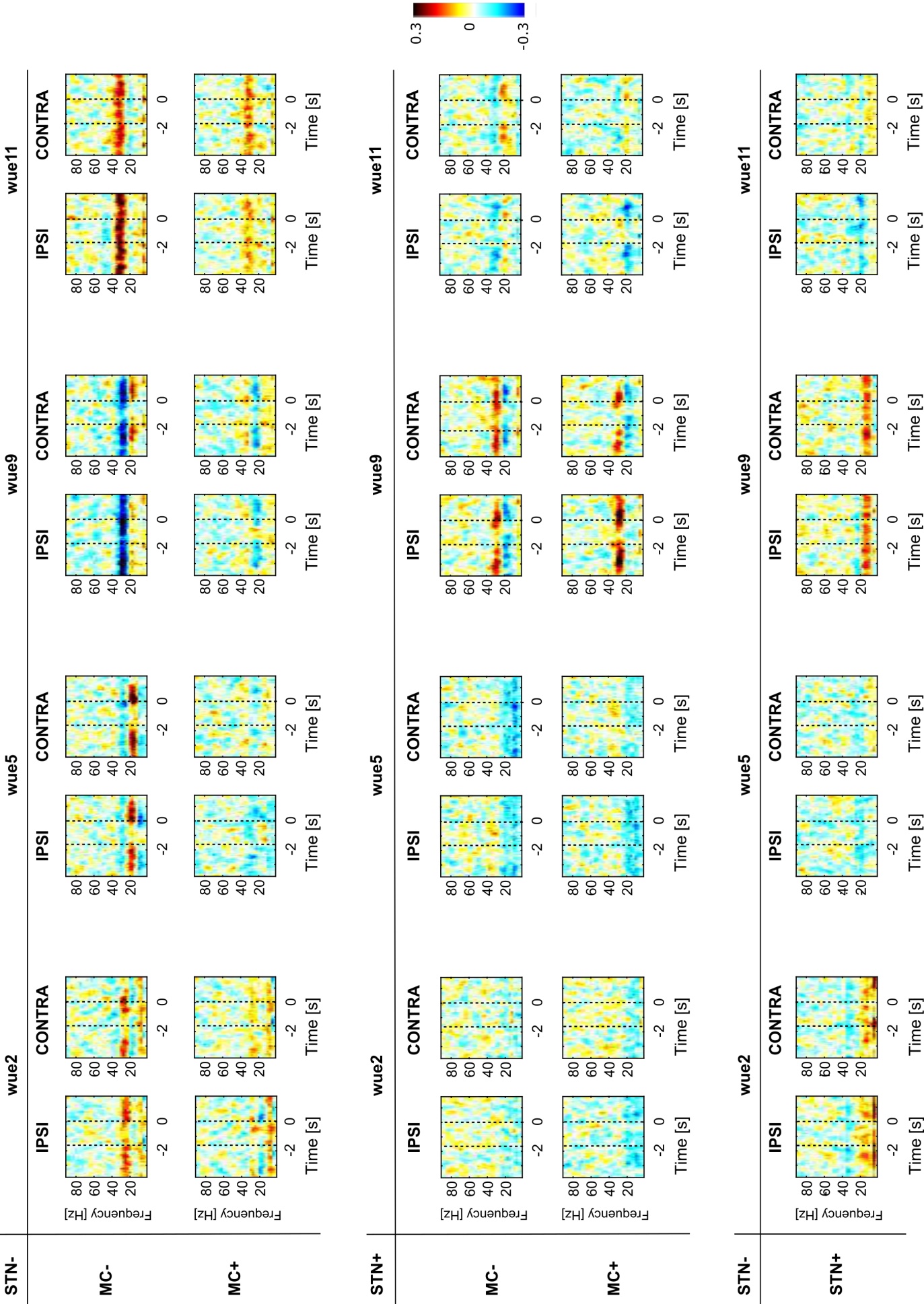
**

***Figure S8.*** *Imaginary part of the coherency analyses. Subcortical- (i.e. iCohSTN–/STN+) and cortical-subcortical imaginary coherency (i.e. iCohSTN–/MC–, iCohSTN–/MC+ and iCohSTN+/MC–, iCohSTN+/MC+) is reported for each subject with respect to the more (STN****–****) and less dopamine-depleted (STN+) subthalamic nucleus. A white color shows lack of coherency. Blue and red dots express the presence of significant coherency among the structures, the intensity of the color express the strength of the coupling. The super-imposed vertical dotted line at 0 s shows the return time. We also indicated with a dotted line at -1.7 s the mean onset time of all trials (see also Figure S1), as a rough suggestion of movement onset time. “IPSI” and “CONTRA” refer to movement performed with the hand ipsilateral or contralateral to the examined subthalamic nucleus. MC = motor cortex, STN = subthalamic nucleus.*
